# Species Invasion History Influences Community Evolution in a Tri-Trophic Food Web Model

**DOI:** 10.1371/journal.pone.0006731

**Published:** 2009-08-24

**Authors:** Akihiko Mougi, Kinya Nishimura

**Affiliations:** 1 Department of Biology, Kyushu University, Fukuoka, Fukuoka, Japan; 2 Graduate School of Fisheries Sciences, Hokkaido University, Hakodate, Hokkaido, Japan; University of Leeds, United Kingdom

## Abstract

**Background:**

Recent experimental studies have demonstrated the importance of invasion history for evolutionary formation of community. However, only few theoretical studies on community evolution have focused on such views.

**Methodology and Principal Findings:**

We used a tri-trophic food web model to analyze the coevolutionary effects of ecological invasions by a mutant and by a predator and/or resource species of a native consumer species community and found that ecological invasions can lead to various evolutionary histories. The invasion of a predator makes multiple evolutionary community histories possible, and the evolutionary history followed can determine both the invasion success of the predator into the native community and the fate of the community. A slight difference in the timing of an ecological invasion can lead to a greatly different fate. In addition, even greatly different community histories can converge as a result of environmental changes such as a predator trait shift or a productivity change. Furthermore, the changes to the evolutionary history may be irreversible.

**Conclusions and Significance:**

Our modeling results suggest that the timing of ecological invasion of a species into a focal community can largely change the evolutionary consequences of the community. Our approach based on adaptive dynamics will be a useful tool to understand the effect of invasion history on evolutionary formation of community.

## Introduction

Understanding the evolution of a biotic community is a major challenge in ecology and evolutionary biology [1], [2], but efforts to do so can help us understand the structures of contemporary ecological communities. One approach is to explore the historical formation of communities [3]–[7]. In this context, research on the ecological and evolutionary formation of communities can potentially help us analyze an emergent problem, the impact of alien species on native communities [8], [9].

The potential key factors affecting the evolutionary formation of communities are immigration, diversification and extinction. Recent experimental studies with micro-organisms and phylogeny analysis have demonstrated several patterns in the evolutionary formation of communities [10], [4], [6]. Meyer and Kassen [6] showed that the evolutionary diversification patterns of a community are influenced by the other community members. Fukami et al. [4] suggested, moreover, that the evolutionary diversification patterns of a community are also greatly sensitive to species immigration history. In spite of such empirical studies showing the importance of the other community members and the timing of historical events, such as species invasion, in community formation, only few theoretical studies have presented an analytical framework for understanding this historical formation of communities, although many theoretical studies have emphasized the importance of evolutionary diversification and/or evolutionary extinction in community formation [11]–[13].

As an initial conceptual exercise, evolutionary biologists often capture coevolutionary phenomena on a dichotomous continuum between pairwise coevolution on one end and diffuse coevolution on the other [14], [15]. Mathematical analysis of coevolution processes and causal mechanisms has focused mainly on pairwise species interactions such as predator–prey and competition [16, but see 11,17–20]. Pairwise coevolution is analytically convenient for understanding community evolution, but it is too simple to capture how historical events such as immigration of species affect the fate of community evolution. In contrast, diffuse coevolution is too complex to analyze, even though it is possible to approach such an analysis [21].

Here we explore how historical events such as ecological or evolutionary species invasions affect the fate of a community. For this purpose, we analyze the eco-evolutionary dynamics of a community resulting from intra- and interspecific competition of one or more consumer species possessing an evolvable trait. For analytical convenience, we consider the community to have at most three trophic levels: an intermediate consumer species that is a native member of the community, a top predator species, and a resource species. Both the predator and resource species invade the community with arbitrary timing in the eco-evolutionary dynamics.

We consider a focal community of one or two consumer species possessing an evolvable trait and analyze the adaptive evolution of the trait, including evolutionary branching, which allows the coexistence of different phenotypes, and the subsequent coevolution of the resulting phenotypes. In the evolutionary dynamics, we consider ecological invasion of the community by a predator or a prey (resource) species. We analyze how the ecological invasion by an alien species influences the evolution of the one or two consumer species, and how that coevolution influences in turn the success of the invasion. We demonstrate that the ecological and evolutionary dynamics result in the development of various communities, depending on the invasion timing and the interactions among species. We also present a potentially new method for examining the evolutionary histories of communities.

## Methods

### Ecological community dynamics

We explicitly describe a part of a food web with at most three trophic levels, a predator species, a resource species, and an intermediate consumer species, competing for the common resources.

The full members explicitly analyzed in the food web are described by the following differential equations:
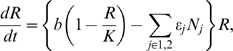
(1a)

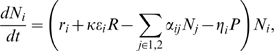
(1b)

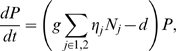
(1c)where *R* is the density of the resource species, *N_i_* is the density of intermediate consumer species *i*, and *P* is the density of the top predator species. The traits of the resource species, the consumer species, and the predator species are *ν*, *u_i_*, and *ω*, respectively. These traits relate to consumption and predation. For example, these traits can be related to size (see below): *ω* may be a trait of the predator that enables it to handle its preferred prey (consumer) size; *u_i_* may be the body size of the consumer, which affects its competitive ability, and *ν* may be the resource size preferred by the consumer.

*ε_i_* is the consumption efficiency of the consumer species, which is a function of the difference between the values of traits *ν* and *u_i_*, and *η_j_* is the predation efficiency of the predator species, which is a function of the difference between the values of traits *u_i_* and *ω*. Predation intensity is assumed to be described on a bidirectional axis of prey vulnerability [22], as is often assumed in predator–prey community modeling (e.g., [23]–[31]. *α_ij_* is the competition coefficient, which is a function of the difference in the resource utilization trait, *u*, between two individual consumers or between consumer species. The competition process is assumed to be asymmetrical [32]–[34]. A possible biological scenario is the following. The predator may have an optimal prey (consumer) size, one that is easier to eat (size-specific predation). In other words, the size of the consumer influences the predation efficiency of the predator. In addition, a consumer with larger body size may be superior in competition with another, smaller consumer species for a resource (whose dynamics is not assumed). However, the size of the consumer can also influence the efficiency with which it can prey upon a second resource species (whose dynamics is assumed) if it also practices size-specific predation on that resource.

*b* and *K* are the intrinsic growth rate and the carrying capacity of the resource species, respectively. *κ* is the conversion rate that relates the consumer's birth rate to resource consumption, and *g* is the conversion rate that relates the predator's birth rate to its consumption of the consumer species. *d* is the mortality rate of the predator. *r_i_* is the intrinsic growth rate of consumer species *i*, which is not affected by consumption of the resource species *R*. We assume that the consumer species can compete for two resources with another consumer species. For simplicity, we do not consider the dynamics of one resource, which influences the intrinsic growth rate of the consumer. We assume that *r_i_* negatively correlates with *u_i_* because a larger body size is costly to maintain (see below for details of the function).

We also assume that the resource utilization trait *u* affects both a consumer's vulnerability to the predator and competition among individual consumers and/or consumer species. Biologically, for example, this assumption may apply to traits such as body size [35], [36]. In other words, predation is size selective and competitiveness is stronger in individuals with larger body size (see the Discussion for cases where these assumptions are broken).

We use the following specific functions in the analysis: bell-shaped functions for the differences in trait values, *ε_i_* = *ε*_0_ exp(−*l*(*u_i_*−*ν*)^2^) and *η_i_* = *η*_0_ exp(−*h*(*u_i_*−*ω*)^2^), where *ε*_0_ (*η*_0_) is the maximum consumption (predation) efficiency of the consumer (predator) species, and *l* (*h*) is the parameter that determines the sensitivity of the consumption (predation) efficiency to the difference in trait values; a sigmoid function of the difference in trait values (competition kernel), *α_ij_* = *c*{1−1/(1+*m·*exp(−*k*(*u_i_*−*u_j_*)))}, where *c* is the maximum value of the mortality rate through competitive interaction, *m* is the parameter that determines the shape of *α_ij_* (concave, linear, or convex), and *k* is the sensitivity of *α_ij_* to the difference in trait values; and a linear function of the trait *u_i_*, *r_i_* = *r*_0_−*λu_i_*, where *r*_0_ is the maximum intrinsic growth rate, and *λ* is the parameter that determines the degree of trade-off.

*m* is a key parameter in this study. This parameter determines the shape of the competition coefficient: concave, linear, or convex, when *u_i_*≃*u_j_*. A concave shape (*m*<1) implies that the competitive advantage of larger size is relatively low and that the competitive disadvantage of smaller size is relatively high. In contrast, a convex shape (*m*>1) implies that the competitive advantage of larger size is relatively high and the competitive disadvantage of smaller size is relatively low. Such effects can greatly influence the consequences of evolutionary trait dynamics.

Possible ecological equilibrium is determined for an ecological community with given parameter values. The ecological dynamics around each equilibrium of a one predator–two consumer system is analyzed in Appendix S1. In our results, the coexistence equilibrium is always locally stable.

### Evolutionary trait dynamics

To describe the evolutionary dynamics of the consumer trait, we use an adaptive dynamics framework, which assumes the separation of dynamics on ecological and evolutionary time scales [24]. For analytical tractability, we assume that the values of the predator and resource species' traits, *ν* and *ω*, are fixed.

The evolutionary dynamics of the consumer trait is based on the occurrence of mutant strategies (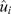) within the resident population of the consumer species and replacement of the resident strategy (*u_i_*) by a mutant strategy as determined by its invasion fitness. Now, consider a rare mutant strategy 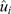 in the resident population with strategy *u_i_*. The mutant increases in number if its growth rate (invasion fitness),
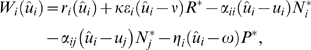
(2)is positive. Otherwise the mutant dies out. 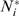, 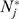, *R* ^*^, and *P* ^*^ represent the equilibrium density of each species.

If phenotypic expressions of mutants are infinitesimally close to those of the resident, the sign of the gradient of the invasion fitness at the resident's trait value,
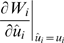
(3)determines whether mutants with smaller or greater trait values can invade, that is, the direction of evolution of the consumer trait in the equilibrium community. If the fitness gradient is positive (negative), a mutant strategy with a higher (lower) trait value than the resident strategy can invade the community and the trait can evolve toward higher (lower) values. During the evolutionary history of a single consumer species, the competitive consumer trait may evolve toward a point where the selection pressure caused by intraspecific competition vanishes. At that point, which is defined as an evolutionary singularity 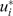, the fitness gradient (eq. (3)) is zero. The condition in which the singularity is an attractor is given by the following local convergence condition,
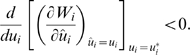
(4)


At the singularity, if
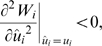
(5)then the selection is stabilizing and the strategy is evolutionarily stable [37]; otherwise, the population undergoes disruptive selection, resulting in evolutionary branching [38]–[40]. On the basis of the fitness gradients (eq. (3)) in the two consumer species, we can describe a directional selection vector of trait evolution in the trait space (*u*_1_, *u*_2_),
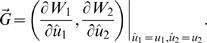
(6)


The evolutionary dynamics of the trait in each consumer species is given by
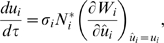
(7)where *τ* is the time domain in which trait evolution occurs, and *σ_i_* ( = 1 in the analysis) is a synthetic constant consisting of the mutation rate and an approximate value for the additive genetic variance [24]. The consumers' traits may continue to coevolve and reach an evolutionary singularity (where both elements in eq. (6) are zero) at which further branching may lead to the development of a diverging community of consumers. In the present study, however, we forgo further evolutionary analysis.

### Eco-evolutionary community dynamics

The evolutionary dynamics (eq. (7)) is coupled with the ecological community dynamics (eqs. (1)). We examine numerically the eco-evolutionary community dynamics. We use a standard adaptive dynamics procedure where after a population of the consumer species is settled to an ecological equilibrium a mutant strategy is introduced into the population and a new ecological equilibrium is calculated. During this cycle of eco-evolutionary dynamics, we introduce a predator and/or a resource species with arbitrary timing when the consumers are at ecological equilibrium. In other words, once the one or two consumer species reach an ecological equilibrium, the introduction of a predator and/or a resource species into the consumer population or community is always attempted. If the invasion of the invader species succeeds, we survey the evolution of the consumer trait (in the one or two consumer species) in the new community and the ecological coexistence of the whole membership of the community.

## Results

### Evolution of consumer community

**Figure 1:**
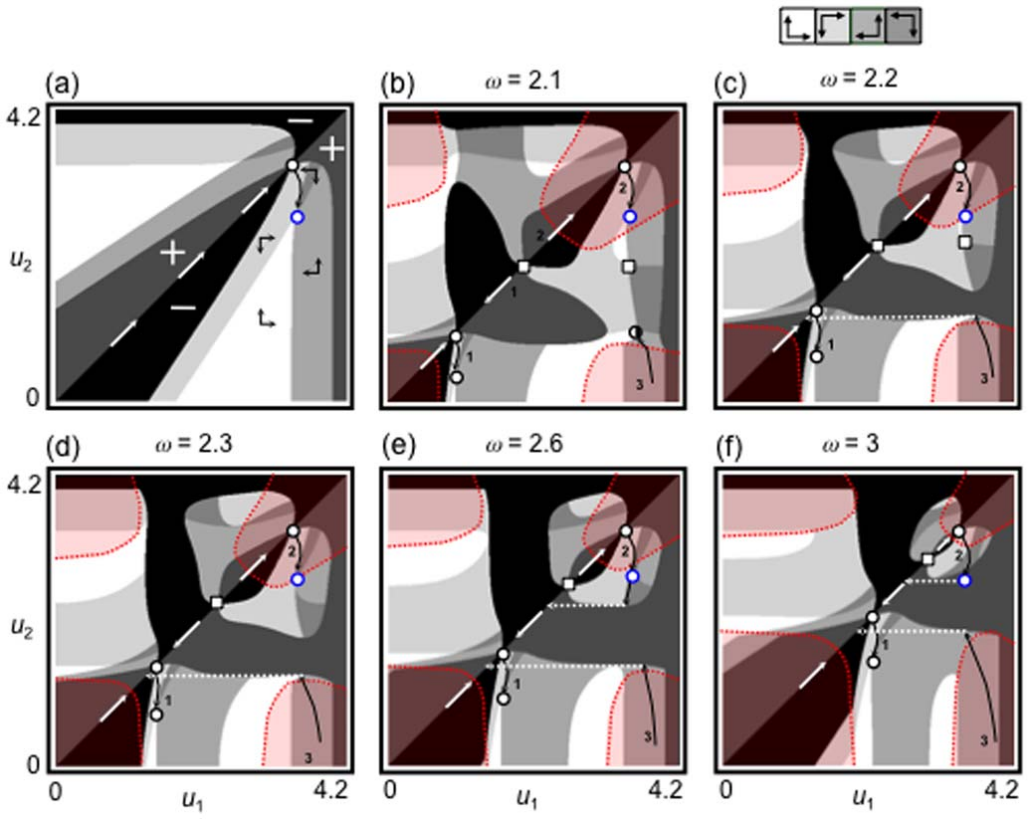
Maps of the evolution of the consumer trait and the community assemblage without (a) or with (b–f) a historical invasion of a predator species. In the maps, eco-evolutionary community development begins with a single consumer species (an arbitrary single point on the diagonal line of each map). Areas above and below the diagonal line exhibit invasibility of mutant traits. The mutant of the black area has a lower invasion fitness and that of the gray area has a higher invasion fitness than the resident (the sign of each is shown in (a), but the signs are omitted in (b–f)). The relative invasion fitness of the mutant determines the evolutionary direction of the consumer trait. The trait evolutionary trajectory of a single consumer species, drawn as white arrows on the diagonal line, leads to diversification by dimorphic trait evolution. The trajectories of trait evolution of the two consumer species after branching are drawn as thin black curved arrows originating at the branching points. The vector space of the consumers' trait evolution in the vicinity of the branching point, which is determined by eq. (6), is indicated by different shading (see the mini-panels at the upper right for the directions of selection). The trait value *ω* of the invading predator species is given above the top of each panel. In panels (b–f), an ecologically invading predator species becomes extinct in the red regions. The black open rectangle is a repellor and the black open circle is an attractor, where branching subsequently occurs. The half-black/half-white circle in (b) represents an evolutionary branching point of consumer species 2 and an ESS of consumer species 1. The blue open circles indicate a singular point that is reached after the branching (if the blue circle is in a region where the predator can exist, no singular point actually exists). The numbers in the panels represent several of the possible evolutionary histories, depending on the value of parameter *ω* (see text). The other parameter values are *g* = 1; *d* = 1; *r*_0_ = 4; *κ* = 1; *λ* = 1; 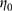 = 2.2; *h* = 1; *c* = 2; *m* = 2; *k* = 5.

In Fig. 1a, we show the typical evolutionary dynamics of the consumer trait in the absence of an ecological invasion by a predator or a resource species. We begin with the evolution of a single consumer species trait by designating *R* ^*^ = *P* ^*^ = 0, and 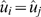 in eq. (2). The evolutionary trajectory is traced by the arrows on the diagonal line in the diagram (see Fig. 1a). The single consumer species evolves to an evolutionary singularity that is also an attractor (open circle on the diagonal line of Fig. 1a). At this singular point, the branching condition holds (i.e., eq. (5) does not hold), leading to evolutionarily dimorphic populations. In our metaphorical interpretation, we assume this process to be one of a diverging community of consumers. After this diversification, the traits of the two consumer species coevolve, following the trajectory from the open circle on the diagonal to the blue open circle in the area of coexistence (see Fig. 1a). Note that we describe the coevolutionary trajectory in the off-diagonal region on only one side of the diagonal because the coevolutionary dynamics are symmetric with respect to the diagonal.

### Major patterns of community evolutionary histories

We consider the impacts of ecological invasions by a predator and/or a resource species on an evolving native community consisting of one or two consumer species. During the consumer species' trait evolution or coevolution, we assume that an ecological invasion of a predator and/or a resource species occurs with arbitrary timing with respect to the trait evolution and evaluate the historical importance of the eco-evolutionary event sequence for the developing community structure.

First, we consider the ecological invasion of a predator into the evolving consumer community (*R* ^*^ = 0, *P* ^*^>0). Figures 1b–f map the trait evolution and evolutionary histories of the community in the face of an ecological invasion by a predator. Notice that the predator species cannot invade in the red regions of the maps; thus, those regions are identical to the corresponding regions on the map shown in Fig. 1a (*P* ^*^ = 0). We find three evolutionary singular points for the trait of a single consumer species (along the diagonal lines on the maps): one evolutionary repellor point (open square) at *u* = *ω*, at which the consumer species suffers the strongest predation pressure from the invading predator, and two evolutionary attractor points (open circles). Thus, two evolutionary histories become possible across the evolutionary repellor (see below).

Now let us consider a scenario of ecological invasion by a predator as the single consumer species is evolving towards higher trait values (see Fig. 1a). If the ecological invasion of the predator occurs when the trait value of the consumer species is located between the evolutionary attractor with a lower value and the evolutionary repellor at the middle of the diagonal (e.g., Fig. 1b), then the evolutionary direction of the consumer trait is reversed and the trait begins to evolve towards the attracter with the lower value. In this ecological invasion, the predator successfully joins the community. If the trait value of the consumer species reaches the lower value evolutionary attractor, then evolutionary branching occurs at that attractor. Throughout the period of evolution and branching, the invading predator can coexist in the community. After this first branching, a one predator–two consumer community emerges and evolves toward a new evolutionary attractor (history 1 in Fig. 1b). For cases of an invading predator with different trait values (different values of *ω*; history 1 in Fig. 1c–f), the evolutionary trajectory is qualitatively the same as in history 1 in Fig. 1b.

In contrast, if the ecological invasion of the predator occurs when the trait value of the consumer species is between the evolutionary repellor and the higher value evolutionary attractor on the diagonal (history 2 in Fig. 1b), either the ecological invasion of the predator does not succeed or, if the ecological invasion initially succeeds, the predator can coexist with the consumer but evolution results in the extinction of the predator. Subsequently, the consumer species attains the evolutionary attractor point with the higher trait value and experiences evolutionary branching without the predator. As a result of the branching, a two-consumer community emerges and evolves toward a new evolutionary attractor; during this period, ecological invasion of the community by the predator species cannot succeed (history 2 in Fig. 1b). See Appendix S2 for details of the evolutionary analysis in the presence of a predator.

The initial community formation pattern depends on the relative values of the predator and consumer traits, *u*−*ω* (Fig. 1b–f). In the numerical examples, when the trait value *ω* of the invading predator is larger relative to the value of *u*, the position of the repellor on the diagonal shifts toward a larger value.

In history 2, after branching the coevolution of the traits of the two consumer species might not (Fig. 1b–d) or might (Fig. 1e, f; see Appendix S3 for the invasion condition of the predator species) allow successful invasion of the community by the predator. If no predator species invades, then the coevolutionary dynamics follows the evolutionary trajectory in Fig. 1a (toward the blue circle). If a predator species can invade (Fig. 1e, f), then the three species can coexist evolutionarily, but the trait coevolution causes one consumer species to become extinct (white dotted arrows extending from history 2 in Fig. 1e, f) and a shift back to evolutionary history 1. This result suggests that the same community can have different evolutionary histories.

We can explore various eco-evolutionary scenarios by using trait coevolution maps (Fig. 1). Among the various possibilities, for instance, let us start from a situation in which the two consumer species already coexist (see the beginning of the history 3 arrow in Fig. 1b, c). The community of coevolving consumers does not initially allow an ecological invasion of a predator species in the trait evolutionary process; however, the traits evolve toward an evolutionary attractor at which the two consumers and the predator can coexist (history 3, Fig. 1b). For larger values of *ω* (e.g., compare Fig. 1c with Fig. 1b), trait evolution from the identical initial condition leads to a different community (history 3 in Fig. 1c): the community coevolves toward a region where one consumer species becomes extinct (white dotted line extending from history 3 in Fig. 1c), causing a shift to history 1 (the same is true for the cases shown in Figs. 1d–f).

### A large evolutionary shift of community

Next, we survey the eco-evolutionary community dynamics with the invasion of both a resource species and a predator species (*R* ^*^>0, *P* ^*^>0). To increase the historical variety of community formation, we also change two ecological factors, the productivity of the basal resource used by the invading resource species, *K*, and the competition asymmetry parameter, *k*.

**Figure 2:**
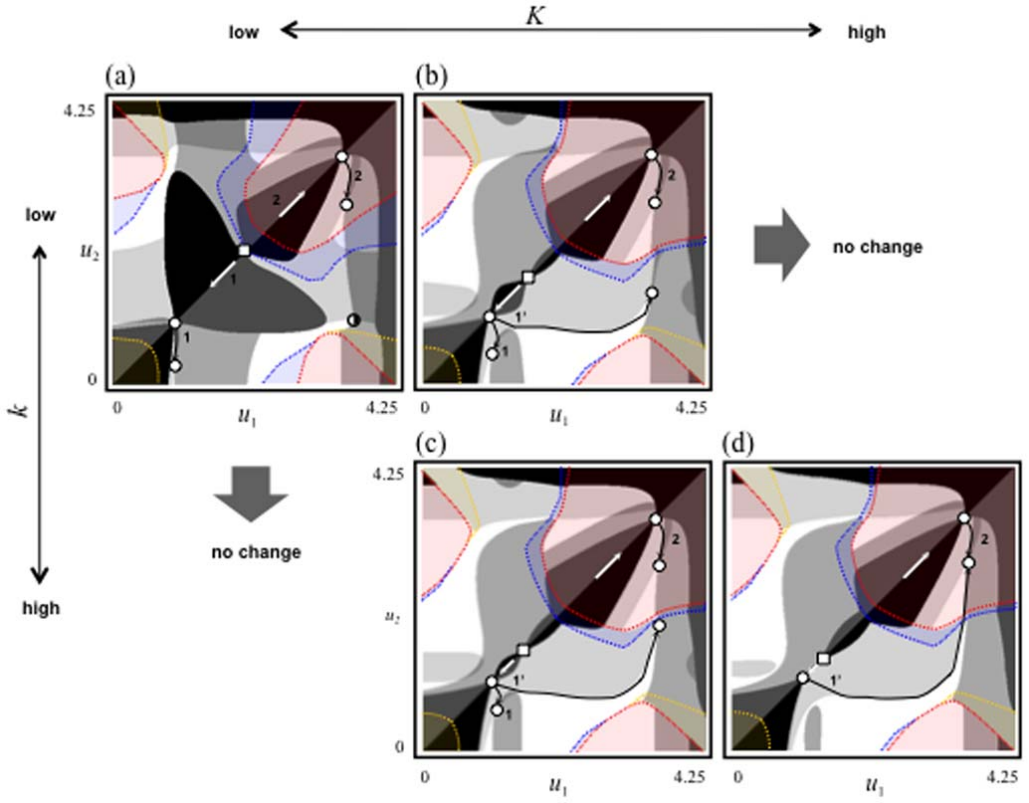
Maps of the evolutionary histories of communities in the face of ecological invasion by a predator and/or a resource species. The red, blue, and yellow regions are those where neither a predator nor a resource species can invade, a resource cannot invade, and a predator cannot invade, respectively. The degree of competitive asymmetry *k* changes from 4.6 (upper panels) to 4.78 (lower panels). The magnitude of productivity *K* changes from 1 to 20 to 25 (left to right). The other parameter values are *g* = 1; *d* = 1; *r*_0_ = 4; *κ* = 1; *λ* = 1; 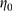 = 2; *h* = 1; *c* = 2; *m* = 1.5; 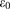 = 2; *l* = 1; *ω* = 2, and *ν* = 2.15. All other information is same as in Fig. 1.

Figure 2 shows evolutionary maps of the consumer trait and the community assemblages with changes in parameters *K* and *k*. When the productivity of the basal resource and the competition asymmetry are both relatively low, two evolutionary histories are possible (Fig. 2a). As the productivity increases, an additional evolutionary path of history 1 becomes possible, depending on the initial conditions, after branching (history 1′ in Fig. 2b, 2c). When the competition asymmetry and productivity are both relatively high, one of the evolutionary paths of history 1 (1 in Fig. 2b) may be eliminated, leaving only the evolutionary path of history 1′, which links to the evolutionary path from history 2 (Fig. 2d). This result suggests that an increase in productivity can lead to the formation of a simple community. This analysis, along with the previous section's analysis results (Fig. 1), also demonstrates that the same community can have different evolutionary histories.

### Parameter dependence

We also investigated to what extent the results obtained depend on the values of the various parameters. In the analysis in the above result sections b and c, we chose the values of parameters *g*, *d*, *r*_0_, *λ*, *κ*, 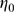, *h*, *c*, 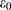, *l*, and *k* so that a predator and/or resource species could successfully invade and coexist with the competing consumer species. We confirmed that this scenario was possible except in the case of much higher or lower values of these parameters. Thus, the precise values of these parameters are not important to the main result.

A particularly key parameter in our result is *m*, which critically determines whether the evolutionary singular points are evolutionarily stable or not. In all of our scenarios, evolutionary branching of the trait of a consumer species is important, but evolutionary branching can occur only if *m* is larger than unity; in contrast, if *m* is smaller than unity, the singular point can be evolutionarily stable (see the derivation in Appendix S2).

## Discussion

We can view a community as having formed by ecological and evolutionary processes acting on the interacting species over many generations. In the coevolutionary process, it is expected that a community will face invasions of a new phenotype or species through events such as evolutionary branching and ecological invasion. Such events create new species interactions, which can change the coevolutionary process of a community and influence the persistence or stability of the community. By understanding the nature of the coevolutionary response of a community to such environmental changes, we expect to find answers to questions about how a community develops during its evolutionary history and how environmental changes such as the introduction of an alien species, for example, via human transport, will influence the native community [9].

We analyzed a model of a coevolving community by focusing on competing consumer species with an evolvable trait and the invasion of an alien predator species and/or resource species into the community. We restricted biological reality for analytical convenience, although we incorporated certain minimum aspects of that reality essential for our purpose.

A major finding of this study is that the presence or absence of a predator species is a key event in community development and that the origin of a new consumer species through evolutionary branching can greatly influence the fate of the subsequent coevolving community facing invasion by a predator. This implies that the evolutionary history of a coevolving community is regulated by the members participating in the formation of the new community. In other words, a community's evolutionary history is highly important in determining its fate. It has been established theoretically that an environmental disturbance during the evolutionary history of a community can regulate the possibility of a genetic polymorphism emerging (‘evolutionary hysteresis’) [41]. In addition, the presence of an exploiter in the evolutionary history of a mutualistic system can immunize the system and prevent its collapse as a result of the subsequent invasion of an exploiter (‘evolutionary immunization’) [18]. The evolutionary immunization effect is similar to our result in that the presence of a predator species in the early evolutionary stage of the community (the predator and a consumer species coexist at the evolutionary branching point with a lower *u* value in Fig. 1b–f) can lead to the evolutionary coexistence of the full community (the predator and two consumer types) (history 1 in Fig. 1b–f). However, the presence of a predator species at a particular point in the evolutionary history of a community, or the evolutionary origin of a new community (i.e., evolutionary branching point), is crucial for the evolutionary consequences of community members after the invasion of the predator, because the evolutionary dynamics from the evolutionary branching point with a higher *u* value where the predator cannot persist can result in the evolutionary extinction of one of the consumer type after the ecological invasion of the predator (history 2 in Fig. 1e,f).

Another related major finding is that the timing of the ecological invasion of a species into a native community is crucially important for the fate of the community. In a coevolving community, a slight difference in the timing of the ecological invasion of an alien species (predator) can result in very different evolutionary histories and community fates (history 1 and 2 in Fig. 1b–f). In addition, we may not be able to distinguish between the possible fates just after a successful invasion, because it is possible for the ecological invasion to succeed initially and for the predator to coexist with the native members of the community evolutionarily, even though on an evolutionary time scale, the invading species becomes extinct and further invasions become impossible (history 1 and 2 in Fig. 1b–f). However, the direction of selection on the interacting traits may provide useful clues as to the eventual fate of the community.

Another key finding is that environmental changes such as a shift in the trait value of the predator (e.g., the change in value from that shown in Fig. 1b or c to that shown in Fig. 1e or f) or a productivity change (e.g., the increase in productivity from that shown in Fig. 2c to that shown in 2d) can potentially cause different community fates (histories 1 and 2 in Figs. 1 and 2) to merge into one evolutionary fate (see the shifts in the evolutionary path of history 2 to history 1, Fig. 1e, f, and history 1 to history 2, Fig. 2d). For example, an evolutionary history in which the evolutionary extinction of one consumer species occurs (history 2 in Fig. 1e, f) may switch to another history in which multiple species can coexist (history 1 in Fig. 1e, f). In contrast, an evolutionary history in which multiple species can coexist (history 1 in Fig. 2c) may merge into a history in which multiple species cannot coexist (history 2 in Fig. 2d). In addition, these evolutionary changes to a community may not be reversible unless another environmental change occurs.

In this study, we assumed specific functions to determine the consequences of interactions between individuals. First, we assumed that the evolvable trait of the consumer affects both its vulnerability to predation and its competitive ability. This assumption, which has some empirical support [35], [36], [42] and which is often made in theoretical studies [26], [43], [44] is critical to our main result, although it is not justified in every natural predator–prey interaction system. We did, however, check that the assumptions that predator–prey and competition interactions, respectively, depend on Gaussian and sigmoid functions were not critical to our main result. The essence of our scenario is that evolutionary multiple attractors can occur in a one predator–one consumer system. Evolutionary multiple attractors can occur even if other interaction functions are used. For example, even if both interaction functions, predator–prey and competition, are Gaussian, evolutionary multiple attractors can occur when the optimum trait values of the predator and consumer are similar. In contrast, if both interaction functions are sigmoidal, evolutionary multiple attractors cannot occur because the trait evolution can escalate as a result of directional selection.

Recently, experimental studies have demonstrated that the existence of a predator species and the immigration history of a species can change the patterns of adaptive radiation in communities of focal competing species [4], [6]. Although the assumption of the mutation with a small phenotypic effect would not be necessarily applied to these microorganisms, our qualitative results also show that the fate of a competing consumer community is greatly changed by the existence of a predator and its invasion timing. Although our model is not practical for prediction of the fate of a natural community, in the future, ecologists will develop methodology not only for the experimental studies and additional theoretical studies but also for studies using molecular phylogeographic approaches [10], [45] to acquire deep understanding of the mechanisms of community evolution.

Our analysis of the coevolution of communities is primitive and overly simple, but a more elaborate analysis is planned for a future paper. However, it provides a theoretical approach to the analysis of complex coevolutionary histories of communities. Theoreticians often analyze extremes because of the potential simplicity of such an analysis, and they seldom deal with intermediate scenarios between the extremes because of their potential complexity. In the present study, the extremes were pairwise coevolution and diffuse coevolution. Pairwise coevolution has already been analyzed well. Our results suggest that we should not ignore intermediate scenarios between pairwise coevolution and diffuse coevolution, although the two extremes are also important subjects of research.

## Supporting Information

Appendix S1 (0.13 MB DOC)

Appendix S2 (0.07 MB DOC)

Appendix S3 (0.08 MB DOC)
