# Supplementary material for: Species Invasion History Influences Community Evolution in a Tri-Trophic Food Web Model
Source: PLoS One. 2009 Aug 24;4(8):e6731. doi: 10.1371/journal.pone.0006731 (PMC2726432; doi:10.1371/journal.pone.0006731)
Supplement: Appendix S1 — (0.13 MB DOC) [file pone.0006731.s001.doc]

# **Appendixes**

We show partial analyses of the system of eqs. 1 (*R* = 0) in the appendixes, because the essence of the behavior of the system does not depend on the resource species but on the predator species, and the entire system with the resource species is analytically complex (however, we were also able to obtain all equilibria analytically in the entire system, including the resource species, and to analyze numerically the local stability of the ecological equilibria and the evolutionary dynamics). In Appendixes S1 and S2, we present ecological and evolutionary analyses, respectively, of the system. In Appendix S3, we analyze the invasion condition for a predator for cases in which an evolutionary singularity exists.

## **Appendix S1:** Ecological analysis

The system of eqs. (1) has seven equilibria *E_i_* (*i* $\in$1, . . . , 7):

(*N*_1_, *N*_2_, *P*) =

*E*_1_: (0, 0, 0),

*E*_2_: (*r*_1_/$\alpha_{11}$, 0, 0),

*E*_3_: (0, *r*_2_/$\alpha_{22}$, 0),

*E*_4_: (*d*/*g*$\eta_{1}$, 0, ($\eta_{1}$*gr*_1_ − *d*$\alpha_{11}$)/$\eta_{1}^{2}$*g*),

*E*_5_: (0, *d*/*g*$\eta_{2}$, ($\eta_{2}$*gr*_2_ − *d*$\alpha_{22}$)/$\eta_{2}^{2}$*g*),

*E*_6_: ((*r*_2_$\alpha_{21}$− *r*_1_$\alpha_{22}$)/($\alpha_{21}\alpha_{12}$ −$\alpha_{11}\alpha_{22}$), (*r*_1_$\alpha_{12}$− *r*_2_$\alpha_{11}$)/($\alpha_{11}\alpha_{22}$ − $\alpha_{21}\alpha_{12}$), 0),

$E_{7}:(\frac{\eta_{2}\left(r_{1}\eta_{2}-r_{2}\eta_{1}\right)+\left(d/g\right)\left(\alpha_{22}\eta_{1}-\alpha_{21}\eta_{2}\right)}{\alpha_{11}\eta_{2}^{2}+\alpha_{22}\eta_{1}^{2}-\left(\alpha_{21}+\alpha_{12}\right)\eta_{1}\eta_{2}},\frac{\eta_{1}\left(r_{2}\eta_{1}-r_{1}\eta_{2}\right)+\left(d/g\right)\left(\alpha_{11}\eta_{2}-\alpha_{12}\eta_{1}\right)}{\alpha_{11}\eta_{2}^{2}+\alpha_{22}\eta_{1}^{2}-\left(\alpha_{21}+\alpha_{12}\right)\eta_{1}\eta_{2}},$ $\frac{\eta_{1}\left(r_{1}\alpha_{22}-r_{2}\alpha_{21}\right)+\eta_{2}\left(r_{2}\alpha_{11}-r_{1}\alpha_{12}\right)+\left(d/g\right)\left(\alpha_{12}\alpha_{21}-\alpha_{11}\alpha_{22}\right)}{\alpha_{11}\eta_{2}^{2}+\alpha_{22}\eta_{1}^{2}-\left(\alpha_{21}+\alpha_{12}\right)\eta_{1}\eta_{2}}$),

Next, we examine the local stability of these equilibria. We obtain the following stability conditions by examining the sign of the real parts of all eigenvalues of the Jacobian matrix at the system equilibrium.

(i) *E*_1_ is always unstable

(ii) *E*_2_ is locally stable if and only if *r*_2_/$\alpha_{12}$< *r*_1_ /$\alpha_{11}$< *d*/*g*$\eta_{1}$ (1a)

(iii) *E*_3_ is locally stable if and only if *r*_1_/$\alpha_{21}$< *r*_2_ /$\alpha_{22}$< *d*/*g*$\eta_{2}$ (1b)

(iv) *E*_4_ is locally stable if and only if 1 *>*$\eta_{1}$(*g*$\eta_{1}$*r*_2_ − *d*$\alpha_{12}$)/$\eta_{2}$(*g*$\eta_{1}$*r*_1_ − *d*$\alpha_{11}$) (2a)

(v) *E*_5_ is locally stable if and only if 1 *>*$\eta_{2}$(*g*$\eta_{2}$*r*_1_ − *d*$\alpha_{21}$)/$\eta_{1}$(*g*$\eta_{2}$*r*_2_ − *d*$\alpha_{22}$) (2b)

(vi) *E*_6_ is locally stable if and only if $r_{1}\left(\eta_{2}\alpha_{12}-\eta_{1}\alpha_{22}\right)+r_{2}\left(\eta_{1}\alpha_{21}-\eta_{2}\alpha_{11}\right)>\left(d/g\right)\left(\alpha_{12}\alpha_{21}-\alpha_{11}\alpha_{22}\right)$ < 0 (3)

(vii) *E*_7_ is locally stable if and only if *A* > *B* > 0,

where *A* =$\left\{\left(\eta_{1}^{2}/N_{2}^{*}+\eta_{2}^{2}/N_{1}^{*}\right)-\left(1/gP^{*}\right)\left(\alpha_{12}\alpha_{21}-\alpha_{11}\alpha_{22}\right)\right\}$and *B =* $\left\{\eta_{2}^{2}\alpha_{11}-\eta_{1}\eta_{2}\left(\alpha_{12}+\alpha_{21}\right)+\eta_{1}^{2}\alpha_{22}\right\}/\left(\alpha_{11}N_{1}^{*}+\alpha_{22}N_{2}^{*}\right)$.

We explicitly obtain a necessary condition for the local stability of *E*_7_,

$\eta_{2}^{2}\alpha_{11}-\eta_{1}\eta_{2}\left(\alpha_{12}+\alpha_{21}\right)+\eta_{1}^{2}\alpha_{22}>0$. (4)

Unfortunately, we cannot determine the stability condition of *E*_7_ fully because it is complex and not analytically tractable. However, we numerically checked that *E*_7_ is always locally stable in the coexistence region of the figures.

We also examine the condition for invasion of a predator into a population of a single consumer species, and into a community of two consumer species. First, we examine the invasion condition of the predator (*dP*/*dt*)(1/*P*) > 0 into a population of a single consumer species (*E*_2_ or *E*_3_) at equilibrium. The condition is

$r_{i}/\alpha_{ii}>d/g\eta_{i}$ (5)

The invasion condition of the predator (*dP*/*dt*)(1/*P*) > 0 into a community of two consumer species at equilibrium (*E*_6_) is

$r_{1}\left(\eta_{2}\alpha_{12}-\eta_{1}\alpha_{22}\right)+r_{2}\left(\eta_{1}\alpha_{21}-\eta_{2}\alpha_{11}\right)<\left(d/g\right)\left(\alpha_{12}\alpha_{21}-\alpha_{11}\alpha_{22}\right)<0$ (6a)

or

$r_{1}\left(\eta_{2}\alpha_{12}-\eta_{1}\alpha_{22}\right)+r_{2}\left(\eta_{1}\alpha_{21}-\eta_{2}\alpha_{11}\right)>\left(d/g\right)\left(\alpha_{12}\alpha_{21}-\alpha_{11}\alpha_{22}\right)>0$ (6b)

In the case of (6a), the conditions necessary to achieve a positive equilibrium of the community consisting of the predator and the two consumers are (4) and (2). In the case of (6b), the conditions are satisfied if the direction of the inequalities, (4) and (2), are reversed. Note that (3) is not satisfied in either case. In other words, when the predator can invade into a community of two consumers, the three species can stably coexist or one consumer species becomes extinct, depending on the initial condition.
