# Supplementary material for: Species Invasion History Influences Community Evolution in a Tri-Trophic Food Web Model
Source: PLoS One. 2009 Aug 24;4(8):e6731. doi: 10.1371/journal.pone.0006731 (PMC2726432; doi:10.1371/journal.pone.0006731)
Supplement: Appendix S2 — (0.07 MB DOC) [file pone.0006731.s002.doc]

## **Appendix S2:** Evolutionary analysis

The evolutionary analysis is applied to the evolutionary dynamics of a single consumer species until another consumer species arises.

We analyze the fitness gradient $\left(\partial W_{i}/\partial\hat{u}_{i}\right)_{\hat{u}_{i}=u_{i}}$ to understand the direction of evolution of the consumer in the presence of a predator (eq. (2) in the text; $N_{j}^{*}$ = *R*^*^ = 0 is used as the mutant fitness of the consumer species). We obtain the fitness gradient,

$\left(\frac{\partial W_{i}}{\partial\hat{u}_{i}}\right)_{\hat{u}_{i}=u_{i}}=r_{i}^{'}\left(u_{i}\right)-\alpha'_{ii}\left(0\right)N_{i}^{*}-\eta'_{i}\left(u_{i}-\omega\right)P^{*}$, (1)

where the prime mark indicates the derivative and the number of marks is the order of the derivative. The sign of (1) determines the direction of evolution. At the evolutionary singularity, the fitness gradient of a competing species is zero.

We analyze the convergence stability to determine whether the singularity is an evolutionary attractor. The singularity is convergence stable if

$\frac{d}{du_{i}}\left[\left(\frac{\partial W_{i}}{\partial\hat{u}_{i}}\right)_{\hat{u}_{i}=u_{i}}\right]_{u_{i}=u_{i}^{*}}$< 0, (2)

where $u_{i}^{*}$ is a singular strategy. We obtain the condition,

, (3)

where $N_{j}^{*}$ and $P^{*}$ are the equilibrium densities at the singular point.

Even if the singular point is an evolutionary attractor, we still cannot determine whether the point is an evolutionary end point. We can ascertain this condition by examining the evolutionary stability, which depends on the second-order derivative of *W_i_*. The singular point is evolutionarily stable (ESS) if

$\left(\frac{\partial^{2}W_{i}}{\partial\hat{u}_{i}^{2}}\right)_{\hat{u}_{i}=u_{i}}<0$. (4)

Otherwise it is evolutionarily unstable, resulting in evolutionary branching. We obtain the ESS condition as follows,

$-P^{*}\left(u_{i}^{*}\right)\eta''_{i}\left(u_{i}^{*}\right)+r_{i}^{' '}\left(u_{i}^{*}\right)<\alpha''_{ii}\left(0\right)N_{i}^{*}$, (5)

If the direction of the inequality is reversed, evolutionary branching occurs. In our specific functions, note that $r_{i}^{' '}$= 0, and $\eta''_{i}$> 0 when . Thus, $\alpha''_{ii}\left(0\right)$< 0 is a necessary condition of evolutionary branching when $u_{i}^{*}$is not close to *ω* . When *m* > 1, this condition is satisfied (not only in the case of *P* but also in the case of *R*, we can also show this condition is likely to cause evolutionary branching ifand ). In the absence of a predator, (2) and (4) become:

$r_{i}^{' '}\left(u_{i}^{*}\right)-\alpha'_{ii}\left(0\right)\frac{r_{i}^{'}\left(u_{i}^{*}\right)}{\alpha_{ii}\left(0\right)}<0$, (6)

$r_{i}^{' '}\left(u_{i}^{*}\right)-\alpha''_{ii}\left(0\right)\frac{r_{i}\left(u_{i}^{*}\right)}{\alpha_{ii}\left(0\right)}<0$. (7)

(7) shows that evolutionary branching can occur when *m* > 1 in our specific functions ($r_{i}^{' '}$= 0).
