# Supplementary material for: Species Invasion History Influences Community Evolution in a Tri-Trophic Food Web Model
Source: PLoS One. 2009 Aug 24;4(8):e6731. doi: 10.1371/journal.pone.0006731 (PMC2726432; doi:10.1371/journal.pone.0006731)
Supplement: Appendix S3 — (0.08 MB DOC) [file pone.0006731.s003.doc]

## **Appendix S3:** Invasion condition for a predator in the case of a singularity

We first examine the condition that a predator can invade a community in which one consumer species is approaching an evolutionary singularity. We obtain the condition,

$\frac{r'_{i}\left(0\right)}{\alpha'_{ii}\left(0\right)}>\frac{d}{g\eta_{i}}$. (1)

We also examine the coexistence condition of the predator and the consumer species in the case that the consumer is approaching the evolutionary singularity. We focus on the condition under which the predator can persist when the consumer is at the singularity. We obtain the condition,

$\frac{r'_{i}\left(0\right)-\left(d/g\eta_{i}\right)\alpha'_{ii}\left(0\right)}{\eta_{i}^{'}\left(u_{i}^{*}-\omega\right)}>0$. (2)

Note that $r'_{i}\left(0\right)$< 0, $\alpha'_{ii}\left(0\right)$< 0, and $\eta'_{i}$> 0 when $u_{i}^{*}$< *ω* (otherwise $\eta'_{i}$< 0).

Next, we examine the condition that a predator can invade a community in which two competitors are approaching a dimorphic evolutionary singularity. We obtain the condition,

$\eta_{1}N_{1}^{*}+\eta_{2}N_{2}^{*}>d$, (3)

where $N_{i}^{*}$ is the equilibrium density of the competitors at the dimorphic evolutionary singularity in the absence of a predator. The equilibrium densities are:

$N_{1}^{*}=A\left(r'_{2}\alpha'_{21}-r'_{1}\alpha'_{22}\left(0\right)\right)$, (4)

$N_{2}^{*}=A\left(r'_{1}\alpha'_{12}-r'_{2}\alpha'_{11}\left(0\right)\right)$, (5)

where *A* = $\alpha'_{12}\alpha'_{21}-\alpha'_{11}\left(0\right)\alpha'_{22}\left(0\right)$. In the specific sigmoid function of the competition coefficient, we have

$A=\left(ckm\right)^{2}\left(-B+C\right)$, (6)

where *B* = 1/(1 + 4*m* + 6*m*^2^ + 4*m*^3^ + *m*^4^) and *C* = 1/(1 + 4*mD* + 2*m*^2^ + 4*m*^2^*D* + 4*m*^3^*D* + *m*^4^). Since *D* = *cosh*(*k*(*u_i_* − *u_j_*)) > 1, *A* < 0. Thus, the terms within the parenthesis in (4) and (5) must be negative for the equilibrium$N_{i}^{*}$ to be positive.

Now, let us return to the analysis of (3). The invasion condition of a predator (3) is likely to be satisfied when$N_{i}^{*}$ >> 0. Thus, the following conditions are necessary for the invasion of a predator:

$\eta_{i}$>>0, (7)

$r'_{i}\alpha'_{ij}-r'_{j}\alpha'_{ii}\left(0\right)$<<0, (8)

$\alpha'_{12}\alpha'_{21}-\alpha'_{11}\left(0\right)\alpha'_{22}\left(0\right)\approx0$. (9)

(8) can be rearranged as

$\frac{\alpha'_{21}}{\alpha'_{22}\left(0\right)}$<<$\frac{r'_{1}}{r'_{2}}$<<$\frac{\alpha'_{11}\left(0\right)}{\alpha'_{12}}$. (10)

Furthermore, by introducing symmetrical parameters, (10) is reduced to

$\alpha'_{12}\alpha'_{21}$<< 1 <<$\alpha'_{11}\left(0\right)\alpha'_{22}\left(0\right)$. (11)

The condition (7) is trivial because it implies that higher predation efficiency is necessary for the invasion of a predator. The other conditions, (9) and (11), are not trivial. The former implies low competitive asymmetry between species. In contrast, the latter implies high competitive asymmetry between species. In other words, the alien predator species cannot invade a community with two coevolving competing species if the two competing species have evolutionary singular strategies that are either too similar or too dissimilar.
